# Therapeutic anticoagulation in patients with acute pancreatitis and splanchnic vein thrombosis: a best evidence topic

**DOI:** 10.1097/MS9.0000000000001440

**Published:** 2023-11-16

**Authors:** Ishtar Redman, Pedram Panahi, Kyriakos Bananis, Panagiotis Drymousis

**Affiliations:** a Department of General Surgery, Ealing Hospital, London North West University Healthcare; b Department of General Surgery, The Hillingdon Hospitals, NHS Foundation Trust, UK

**Keywords:** acute pancreatitis, anticoagulation, bleeding complications, rates of recanalization, splanchnic vein thrombosis

## Abstract

A best evidence topic in general surgery was written according to a structured protocol. The clinical question addressed was: in adult patients with splanchnic vein thrombosis in acute pancreatitis, would administration of therapeutic anticoagulation be advisable considering the rates of vessel recanalization and bleeding complications? Four hundred twenty-four papers were found on Ovid Embase and Medline whilst 222 were found on PubMed using the reported literature search. From these, five articles represented the best evidence to the clinical question. The authors, publication dates, countries, patient groups, study outcomes, and results of these papers were tabulated. There were three systematic reviews with meta-analyses, one systematic review without meta-analysis and one randomized, retrospective study. The authors conclude that among patients with splanchnic vein thrombosis in the context of acute pancreatitis, therapeutic anticoagulation improved the rates of recanalization without increasing the risk of bleeding complications. However, there remains a need for randomized studies to address this clinical dilemma to further increase the quality of available evidence.

## Introduction

Highlights

- Splanchnic vein thrombosis is common in acute pancreatitis.
- Anticoagulation improves vessel recanalization rates with no increase in bleeding.
- A patient individualised anticoagulation policy should be adopted.

The management of splanchnic vein thrombosis in patients with acute pancreatitis remains a controversial topic with a paucity of randomized evidence available. At present, there is little consensus on whether patients with splanchnic vein thrombosis in the context of acute pancreatitis should receive therapeutic dose anticoagulation. A best evidence topic was constructed according to a structured protocol.

## Clinical scenario

A 35-year-old man presents to accident and emergency with a 2-day history of nausea, vomiting, anorexia, and epigastric pain on a background of alcohol excess. His biochemistry reveals a serum amylase of 2000 U/l (Normal range: 30–110 U/L), a C- reactive protein of 78 mg/l (normal range: 3–10 mg/l) and a white cell count of 14×10^9^/l (normal range: 4.0–11.0×10^9^/l). A diagnosis of acute pancreatitis is made. Seventy-two hours following admission, a contrast enhanced computed tomography of his abdomen confirms the diagnosis but also reveals evidence of splanchnic vein thrombosis.

You recall the ongoing controversy around the best management options: therapeutic anticoagulation (AC) versus conservative management. Unsure which is in the best interest of the patient, you resolve to check the literature for evidence.

## Three-part question

In (adult patients with splanchnic vein thrombosis in acute pancreatitis), would (administration of therapeutic AC) be advisable considering the (rates of vessel recanalization and bleeding complications)?

## Search strategy

Electronic searches were performed on both Medline (1946 to September 2023) and Embase (1974 to September 2023) using the OVID interface as well as Medline using the PubMed interface. The search terms were as follows:

(Pancreatitis OR Acute Pancreatitis) AND (Splanchnic vein OR mesenteric vein OR portal vein OR splenic vein OR hepatic vein OR lienal vein) AND (thrombus OR clot OR thrombosis) AND (anticoagulation OR anticoagulation OR DOAC OR NOAC OR heparin OR warfarin OR vitamin K antagonist*).

These keywords were searched in the subject headings, in title and in abstract. Where possible, the results were limited to English articles, human studies, and adult population. All reference lists of the included papers were also screened to identify any pertinent studies. The results were current as of September 2023.

## Search outcome

Four hundred twenty-four papers were found using the reported search on Embase (362) and Medline (62) using the Ovid interface whilst 222 were found on Medline using the PubMed interface. Three independent investigators screened the articles and selected the best evidence for this review. There were no discrepancies. Case reports, case studies, editorials, duplicates, literature reviews, single-centre studies, and studies with paediatric patients were excluded.

**Figure 1:**
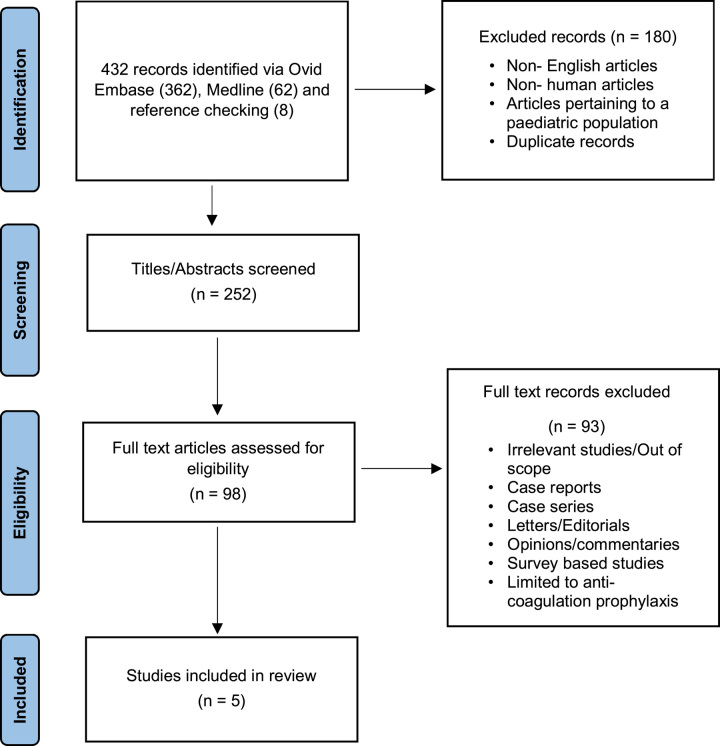
PRISMA flow chart for Ovid search (PubMed flow chart not included in this figure).

The best evidence to answer our clinical question consisted of five studies whose primary outcomes were the rates of vessel recanalization and bleeding complications in patients who received therapeutic AC compared to those who did not (N_AC). The complete literature search is outlined in Appendix 1. An example of the screening and eligibility assessment process for the search results obtained from the Ovid interface is outlined in the PRISMA diagram below (Fig. 1).

## Results

The results of this review can be found in (Table 1). This contains an overview of the most pertinent and highest quality evidence available assessing the outcomes of anticoagulating patients with splanchnic vein thrombosis in the context of acute pancreatitis. The table was structured in line with the guidance stipulated by the *International Journal of Surgery* ^6^, highlighting the key results, analytical outcomes, and study limitations.

**Table 1:** Study characteristics.

Article	Author, date, journal, country	Study type and level of evidence	Patient group	Outcomes	Key results	Comments
Current practice of anticoagulant in the treatment of splanchnic vein thrombosis secondary to acute pancreatitis^1^	Norton W *et al.*, (2020), Hepatobiliary & Pancreatic Diseases International, Singapore	Systematic review (Level II)	Anticoagulated (AC) = 92 Non-anti-coagulated (N_AC) = 106	Vessel recanalization at follow up scan bleeding complications	AC: 14% and N_AC: 11% AC: 16% N_AC:5%	No RCTs included: (nine case reports, two case series, five retrospective single-centre studies). Excluded patients with chronic pancreatitis or those with pancreatitis after surgical interventions: pancreatic resection or transplant. Studies too heterogeneous to allow for meta-analysis
Use of therapeutic anticoagulation in splanchnic vein thrombosis associated with acute pancreatitis: a systematic review and meta-analysis^2^	Chandan S *et al.*, (2021), Annals of Gastroenterology, Greece	Systematic review and Meta-analysis (Level I)	Anticoagulated (AC) =103 Non-anti-coagulated (N_AC) =136	Pooled rates of vessel recanalization Difference between the two groups Pooled rates of bleeding complications Difference between the two groups Pooled rates of collateral formation Difference between the two groups Pooled death rates Difference between the two groups	AC: 53/103; 51.5% 95% CI: 35.5–67.1 *I* ^2^ = 52% N_AC: 40/136; 28.6% 95% CI: 18.6–41.3 *I* ^2^ =38% RR=1.6 95% CI: 1.17–2.27 *I* ^2^=0%; *P*=0.004 AC: 21.2% 95% CI: 14–30.6 *I* ^2^=0% N-AC 11% 95% CI: 6.5–17.9 *I* ^2^=0% RR =1.95 95% CI: 0.98–3.88 *I* ^2^=0% *P*=0.06 AC= 43.3% 95% CI: 26.1–62.3 *I* ^2^ =61% N_AC=46.2% 95% CI: 31.3–61.8 *I* ^2^ =26% RR=1.24 95% CI: 0.75–2.05 *I* ^2^ =24% *P*=0.4 AC= 12.6% 95% CI: 7.5–20.4 *I* ^2^=0% N_AC=6.8% 95% CI: 3.5–12.8 *I* ^2^=0% RR =2.02 95% CI: 0.85–4.8 *I* ^2^=0% *P*=0.1	No RCTs included: (six retrospective studies, one prospective multicentre study). Large sample sizes allowing for pooling of data and meta-analysis. Excluded patients with chronic pancreatitis or those receiving prophylactic doses of anti-coagulation. Demonstrated that the difference in recanalization rates between the two groups was statistically significant but the difference in bleeding complications was not. Unable to assess publication bias as study number <10.
A selective anticoagulation policy for splanchnic vein thrombosis in acute pancreatitis is associated with favourable outcomes: experience from a UK tertiary referral centre^3^	Thejasvin K *et al.*, (2022), HPB (Oxford), England	Randomised, retrospective, multicentre trial (Level II)	Anticoagulated (AC) =74 Non-anti-coagulated (N_AC) =35	Vessel recanalization at follow up scan Bleeding complications Venous collateral formation rates (by sub-group) Mortality	Portal/Mesenteric_AC: 42/63, 66.7% Progressive splenic vein_AC 2/11, 18.2% N_AC 11/35, 31.4% *P*=0.003 AC: 5/74, 6.8% N-AC: 0/35 *P*=0.4 Persisting thrombus 24/32; 75% Recanalization 8/32; 25% Progressive splenic vein_AC 2/11 N_AC 12/35 Inpatient mortality 8/401; 2% Overall mortality 66/401; 16.5%	Excluded patients with evidence of chronic pancreatitis, those undergoing abdominal surgery at the time of acute pancreatitis and patients with a history of cirrhosis/thrombotic disorders. Patients with evidence of isolated, nonpropagating splenic vein thrombus treated with prophylactic doses of anticoagulation. Rates of recanalization and bleeding complications separated by anatomical locations within the splanchnic system
Therapeutic anticoagulation for splanchnic vein thrombosis in acute pancreatitis: a systematic review and meta-analysis^4^	Sissingh NJ *et al.*, (2022), Pancreatology, Switzerland	Systematic review and Meta-analysis (Level II)	Anticoagulated (AC) =70 Non-anti-coagulated (N_AC) =83	Vessel recanalization at follow up scan Bleeding complications Formation of varices/collaterals/cavernoma Mortality	AC: 25/70: 36% N_AC: 17/83: 20% Absolute risk difference =0.09 95% CI: −0.11–0.28 *I* ^2^=48% AC: 17/94: 18% N_AC 8/104: 8% Absolute risk difference for haemorrhage =0.03 95% CI: −0.06–0.12 *I* ^2^=2% AC=45/89; 59% N_AC= 42/74; 57% Absolute risk difference in varices/collaterals/cavernoma between AC and N_AC = −0.03 95% CI: −0.19–0.12 *I* ^2^=0% AC= 3/45; 7% N_AC= 3/51; 6% Absolute risk difference in mortality = 0.02 95% CI: −0.08–0.12 *I* ^2^=0%	No RCTs included: (seven retrospective cohort studies). Variable data available on specific outcomes within the included studies: Recanalization rates and bleeding complications reported in six of the seven studies. Formation of varices/collaterals/cavernoma rates available for 5/7 studies. Mortality figures available in 3/7 studies. Included studies reporting on adult patients with acute pancreatitis, splanchnic vein thrombosis and at least one clinical outcome of interest. Overall risk of bias considered moderate due to risk of confounding within all studies
Incidence and treatment of splanchnic vein thrombosis in patients with acute pancreatitis: a systematic review and meta-analysis^5^	Anis FS *et al.*, (2022), Journal of Gastroenterology and Hepatology Australia, 2022	Systematic review and Meta-analysis (Level II)	Anticoagulated (AC) =264 Non-anticoagulated (N_AC) =679	Vessel recanalization at follow up scan Bleeding complications Mortality	AC: Recanalization more likely to occur in the anticoagulation-treated than in the untreated group OR 0.51 95% CI: 0.31–0.83 *P*=0.007 *I* ^2^=0% OR 2.27 95% CI: 0.81–6.37 *P*=0.12 *I* ^2^ =33% OR 2.37 95% CI: 0.86–6.52 *P*=0.10 *I* ^2^=0%	No RCT’s included: (15 retrospective and three prospective studies) Moderate risk of bias due to risk of selection bias and lack of randomisation/ standardized treatment protocols. Included studies reporting on adult patients with acute pancreatitis, splanchnic vein thrombosis and at least one clinical outcome of interest. Moderate bias reported in 14/18 studies due to retrospective study designs, small sample sizes and limited data on anti-coagulation allocation

## Discussion

Acute pancreatitis is a relatively common presentation to the acute surgical take and is associated with significant morbidity, mortality, and hospitalisation costs^7^. Current literature on temporal trends reveals that the incidence of acute pancreatitis has been increasing globally over the past 20 years, with average annual per cent changes of 3.67 and 2.77% across North America and Europe, respectively^8^. In the United Kingdom, the incidence of acute pancreatitis is estimated at 48.2 per 100 000, per year^9^. Multiorgan failure, localised collections, and thrombosis of the splanchnic vessels are well recognised complications of acute pancreatitis^10^. Anatomically, the splanchnic venous system includes the splenic, portal, superior, and inferior mesenteric veins, with the splenic vein being the most common site for thrombosis in the context of acute pancreatitis^11^.

Although the overall incidence of acute pancreatitis related splanchnic vein thrombosis is reported at 15%^5^, many authors believe that this figure is likely an underestimate, as most cases are identified incidentally on cross-sectional imaging and patients classified with mild to moderate disease often forego imaging entirely.

In the context of acute pancreatitis, spontaneous recanalization of the splanchnic venous system has been reported to occur in approximately one third of patients^2^. Nonetheless, the importance of early recognition and timely intervention cannot be understated; if left untreated, splanchnic vein thrombosis may result in mesenteric infarction, hepatic ischaemia, and portal hypertension^12^.

Outside of the clinical context of acute pancreatitis, thrombosis of the splanchnic venous system has been well documented in patients with liver cirrhosis and other prothrombotic disease states such as myeloproliferative disorders, inherited thrombophilia, and solid organ malignancies^13^. Comparative literature addressing AC, recanalization rates, and bleeding complications in this cohort of patients is low strength. They primarily include observational studies on patients with liver cirrhosis-related splanchnic vein thrombosis receiving low‑molecular‑weight heparin, Vitamin K antagonists and more recently, direct oral anticoagulant agents (DOACs)^14^. Such patients are often medically complex and are managed by a multidisciplinary team of haematologists, gastroenterologists/hepatologists, interventional radiologists, and hepatobiliary surgeons. Current treatment guidelines on this topic reflect the sparsity of high-quality evidence, yet expert consensus recommends therapeutic doses of AC for cirrhotic patients with splanchnic vein thrombosis^15^. In some specialist tertiary centres, novel approaches such as catheter-directed thrombolysis, mechanical thrombectomy, and transjugular intrahepatic portosystemic shunt (TIPS) implantation have been described as alternative treatments to systemic AC in a select few patients; however, studies on these modalities have reported non-negligible bleeding risks primarily due to intra-abdominal or gastrointestinal bleeding^16,17^.

Management of acute pancreatitis related splanchnic vein thrombosis in patients without any of above-mentioned co-morbidities is complicated by a lack of high-quality clinical trials often resulting in significant variation in treatment protocols amongst clinicians. Considering this, we conducted a review of the best evidence topic for this specific cohort of patients.

In 2020, Norton *et al.* ^1^, conducted a systematic review of 16 publications whose primary outcomes were vessel recanalization and bleeding complications. Of the 198 patients included in the study, 92 (46.5%) received anticoagulation therapy. The rates of recanalization in the treated and nontreated groups were 14 and 11% in contrast to the rates of bleeding complications, quoted as 16 and 5%, respectively. The authors highlighted a major limitation within their study which concerned the clinical and statistical heterogeneity amongst the studies which precluded them from conducting a meta-analysis. Due to the limited availability of literature at the time of this review, the authors were unable to recommend one treatment over the other, but commented on the need for high-powered, randomized controlled trials in specialist tertiary centre settings.

Chandan *et al.* ^2^, performed a systematic review comparing the clinical outcomes of patients with acute pancreatitis and splanchnic vein thrombosis who received therapeutic anticoagulation to those who did not. The authors of this review were able to perform a meta-analysis to calculate the relative risk (RR) of vessel recanalization and bleeding complications in addition to the secondary outcomes of collateral formation and mortality. Their study revealed a significantly higher proportion of patients with vessel recanalization in the anticoagulated group with a risk ratio (RR) of 1.6 (95% CI: 1.17–2.27; *I* ^2^=0%; *P*=0.004) without an associated difference in the relative risk (RR) of bleeding complications, collateral formation, or mortality. Like Norton *et al.*, the authors were able to demonstrate that therapeutic AC resulted in greater rates of vessel recanalization without a statistically significant difference in bleeding rates between the two groups (RR 1.95, 95% CI: 0.98–3.88; *I* ^2^=0%; *P*=0.06.) However, the authors emphasised that despite this, there was a demonstrable trend towards increased bleeding in patients who received anticoagulation, ultimately concluding that in the absence of contraindications, therapeutic anticoagulation should be considered.

In the study conducted by Thejasvin *et al.* ^3^, greater emphasis was placed on identifying the incidence, risk factors and treatment outcomes of patients with splanchnic vein thrombosis. Their study sub-categorised the rates of recanalization by vessel subtypes, demonstrating superior rates of recanalization for portal vein thrombus (66.7%) compared to splenic vein thrombus (18.2%). Five of their 74 anticoagulated patients developed bleeding complications: two sources of haemorrhage from the splenic artery, one from the gastroduodenal artery, and two from their upper gastrointestinal tract. Zero of the 35 nonanticoagulated patients went on to develop bleeding complications. From this, the authors concluded that therapeutic dosage of anticoagulation was not associated with a significantly higher rates of haemorrhagic complications, reporting a nonsignificant difference in bleeding complications between those that did and did not receive anticoagulation.

Sissingh *et al.* ^4^, conducted a systematic review and meta-analysis of seven retrospective cohort studies. The primary outcomes of their study were similar to those reported on by Chandan *et al.*, and described rates of recanalization, haemorrhage, and mortality in addition to recurrent venous thromboembolism, development of varices, collaterals or cavernomas. The authors reported pooled recanalization rates in the anticoagulated group of patients at 36%, in contrast to a rate of 20% in patients who did not receive therapeutic AC. This corresponded to absolute risk differences of 9% (95% CI: −0.11–0.28; *I* ^2^=48%) for recanalization and 3% (95% CI: −0.06–0.12; *I* ^2^=2%) for haemorrhagic complications. Interestingly, the reviewers purported that the 3% absolute risk difference for haemorrhagic complications was likely an underestimate secondary to selection bias whereby the perceived increased bleeding risk in patients with acute pancreatitis, impacted the decision not to treat with therapeutic doses of AC. As such, the authors were not able to demonstrate that therapeutic anticoagulation improved rates of recanalization compared to no therapeutic anticoagulation.

In 2021, Anis *et al.* ^5^, conducted a systematic review and meta-analysis of 18 studies. This study mirrored the review done by Thejasvin *et al.*, where primary outcomes included identifying the incidence of splanchnic vein thrombosis in patients with acute pancreatitis and assessing the effects of therapeutic AC. Of the 943 patients included: 264 (28%) received AC, recanalization rates were higher in the anticoagulated group than the untreated group, odds ratio (OR) 0.51 (95% CI: 0.31–0.83, *I* ^2^=0%, *P*=0.007). Furthermore, like Chandan *et al.* ^5^, this study found no difference in haemorrhagic complications in relation to the use of AC (OR 2.27, 95% CI: 0.81–6.37, *I* ^2^=33%, *P*=0.12), ultimately concluding that treatment with therapeutic doses of AC increases recanalization rates without increasing rates of haemorrhagic complications.

Intuitively, one would expect that administering therapeutic doses of AC would incur an increased bleeding risk. However, three of the five studies (Chandan *et al.* ^2^, Thejasvin *et al.* ^3^, and Anis *et al.* ^5^), all reported an improvement in the rates of recanalization in patients who received therapeutic AC without an associated increase in bleeding complications. One potential explanation for this phenomenon was hypothesised by Sissingh *et al.*, who suggested that treatment with therapeutic AC might prevent thrombosis propagation, ultimately reducing portal pressure and subsequently decreasing the risk of haemorrhage. Supporting this theory, are the findings of an additional meta-analysis by Valeriani *et al.* ^14^, who reported lower rates of bleeding complications in patients treated with therapeutic AC (9%) compared to untreated patients (16%). Of note, their study differed from those reviewed within this article and focused on patients with underlying liver cirrhosis, myeloproliferative neoplasms, and solid organ malignancies with or without thrombophilia. Norton *et al.* ^1^ highlighted the overall lack of randomized studies addressing this question whilst the meta-analysis conducted by Sissingh *et al.* ^4^, concluded that the lack of data was insufficient to conclude if therapeutic AC was beneficial.

Ultimately, the heterogenous results in the above studies, in addition to the overall paucity of high-quality evidence on this topic, highlights the need for clinicians to adopt a selective AC policy for patients with splanchnic vein thrombosis in the context of acute pancreatitis. Therapeutic AC will result in superior recanalization rates; however, the risk of bleeding complications is not negligible.

## Clinical bottom line

On the basis of this analysis, we can conclude that in adult patients with splanchnic vein thrombosis in the context of acute pancreatitis, therapeutic AC results in superior canalisation rates; however, the risk of bleeding remains an area of concern. The decision to actively treat splanchnic vein thrombosis must be carefully balanced against this risk and a selective patient specific anticoagulation policy should be adopted. Given the well-established complications associated with splanchnic vein thrombosis in conjunction with the potential bleeding risks posed by therapeutic doses of anticoagulation, this question remains a clinically relevant one. To remedy this area of uncertainty, high-powered, randomized controlled, multicentred trials with large patient numbers should be undertaken.

## Ethical approval

Ethics approval was not required for this review.

## Consent

Informed consent was not required for this review.

## Sources of funding

No funding to declare.

## Author contribution

I.R.: generated the research proposal, conducted the literature search, and wrote the manuscript; P.P.: reviewed the literature search and methodology and assisted in writing the paper; K.B.: reviewed the literature search and methodology and assisted in writing the paper; P.D.: supervising consultant and manuscript review.

## Conflicts of interest disclosure

There are no conflicts of interest.

## Research registration unique identifying number (UIN)

Not applicable.

## Guarantor

Dr Ishtar Redman.

## Data availability statement

Not applicable.

## Provenance and peer review

Not applicable.
